# International University Students’ Pre-Travel Preparation, Knowledge and Practices towards Travel Health in Thailand: A Nationwide Cross-Sectional Study

**DOI:** 10.3390/tropicalmed8060322

**Published:** 2023-06-15

**Authors:** Sawettachai Jaita, Phimphan Pisutsan, Saranath Lawpoolsri, Amornphat Kitro, Chatporn Kittitrakul, Teera Kusolsuk, Supitcha Kamolratanakul, Poom Chompoosri, Gerard T. Flaherty, Jittima Dhitavat

**Affiliations:** 1 Thai Travel Clinic, Hospital for Tropical Diseases, Faculty of Tropical Medicine, Mahidol University, Bangkok 10400, Thailand; sawettachai@thaitravelclinic.com; 2 Department of Clinical Tropical Medicine, Faculty of Tropical Medicine, Mahidol University, Bangkok 10400, Thailand; chatporn.kit@mahidol.ac.th (C.K.); supitcha.kam@mahidol.edu (S.K.); jittima.dhi@mahidol.ac.th (J.D.); 3 Department of Tropical Hygiene, Faculty of Tropical Medicine, Mahidol University, Bangkok 10400, Thailand; 4 Department of Community Medicine, Faculty of Medicine, Chiang Mai University, Chiang Mai 50200, Thailand; amornphat.kit@cmu.ac.th; 5 Department of Helminthology, Faculty of Tropical Medicine, Mahidol University, Bangkok 10400, Thailand; teera.kus@mahidol.ac.th; 6 School of Medicine, Mae Fah Luang University, Chiang Rai 57100, Thailand; poom.cps@gmail.com; 7 School of Medicine, University of Galway, H91 TK33 Galway, Ireland; gerard.flaherty@universityofgalway.ie; 8 School of Medicine, International Medical University, Kuala Lumpur 57000, Malaysia

**Keywords:** international university students, Southeast Asia, Thailand, young adult travellers, motorcycle, sociocultural factors, online survey, emergency services number

## Abstract

International university students are vulnerable travellers due to their unpredictable schedules and lifestyles. As Thailand continues to see an increase in international students, evaluating their pre-travel preparation and preventive behaviours is crucial to identify areas for improvement. For this purpose, an online survey focusing on pre-travel preparation, knowledge and preventive practices related to travel health was distributed to 324 eligible international students from 14 Thai universities, with the majority being from Asia and Oceania (79.0%; *n* = 256). The results showed that half of the respondents (53.7%; *n* = 175) received professional pre-travel advice, mainly because of the mandatory health examination and vaccination requirements of the host university. The study also revealed inadequate knowledge about infectious and non-infectious health risks, with only one-third being aware that Japanese encephalitis is transmitted by mosquito bites, and less than half of the students recognising Thailand’s emergency services number. Poor preventive practices were also observed, with less than half of those with new sexual partners consistently using condoms and less than half of those riding motorcycles always wearing helmets. These findings highlight the need for a new strategy to improve the standard of travel health preparation among this group of young adult travellers, particularly those from resource-limited countries.

## 1. Introduction

After the emergence of globalisation, international student mobility has consistently increased over the last two decades. The annual numbers of internationally mobile students have doubled from 2.2 million in 1998 to over 5.6 million in 2018 [1]. Despite the transient decline in international student mobility growth from the COVID-19 pandemic [2], the trend is expected to promptly return to its pre-pandemic state. Whilst Western countries have always been the traditional preference for higher education, several countries in Asia-Pacific regions have recently demonstrated an increasing share of international students [3]. Thailand has been striving to internationalise its higher education system since the 1990s [4]. This effort has placed Thailand as the third most popular educational hub in Southeast Asia, following Singapore and Malaysia [5]. In the last decade, the number of international students in Thailand has nearly doubled, with over 27,128 currently enrolled [6].

International students are considered a high-risk category of travellers due to their shared characteristics with travellers visiting friends and relatives (VFR), which include an underestimated risk perception, poor preventive practices and failure to seek pre-travel preparation [7,8,9]. Nevertheless, there are also distinctive features that differentiate international students from other traveller groups. For instance, the demanding schedules and unpredictable lifestyles of university students can pose a challenge to receiving appropriate pre-travel preparation. Moreover, a considerable number of students may indulge in alcohol and recreational drugs while studying abroad, leading to transport-related injuries and unsafe sex [10]. Furthermore, some students with financial constraints may not have access to appropriate travel vaccines or first-aid medication since these preventive measures may not be subsidised by their universities [11].

Based on the previous literature, international students’ adherence to pre-travel advice varied widely across different student groups. For instance, surveys conducted among Western international students have reported uptake rates of professional pre-travel consultation ranging from 24% to 93% [8,9,12,13,14]. A few studies among Asian students have revealed significantly lower uptake rates ranging from 0% to 25% [15,16,17]. Additionally, students in healthcare disciplines have exhibited a greater adherence to professional pre-travel advice than those from other fields [13]. However, despite the availability of professional consultation, the majority of students still opted to seek travel health information through non-professional sources such as the Internet, friends and family or guidebooks [12,17,18]. This practice can be problematic as the information obtained from these sources may not always be reliable, leading to inappropriate travel health practices [12]. Research has also indicated an insufficient level of knowledge and preventive behaviours related to travel health among this group of travellers [8,12,17]

Regarding the prevalence of travel-related illnesses among international university students, previous surveys have reported a rate of 17–32% [12,13,17], with travellers’ diarrhoea being the most common, affecting approximately 30–45% of students [13,19]. Other reported health problems included respiratory illnesses, acute febrile illness, dermatological diseases, sexually transmitted diseases, mental health problems, traffic injuries, physical violence and needle-stick injuries while working in healthcare facilities [12,13,14,19,20].

Despite the increasing number of international students and concerns regarding their travel health preparedness, research on this vulnerable group of travellers remains limited. Most previous studies have focused on Western students or those in healthcare disciplines, leaving a significant gap in knowledge about non-Western and non-healthcare students’ travel health behaviours. Moreover, there is still a scarcity of information on students’ knowledge and preventive practices regarding travel-related health issues. The primary objective of this study aimed to explore characteristics of pre-travel preparation, knowledge and preventive practice among international university students enrolled in Thai institutions. The secondary objective was to survey the prevalence of travel-related illnesses during their studies in Thailand.

## 2. Materials and Methods

### 2.1. Study Design and Participant Selection

A multisite cross-sectional study was carried out among international university students enrolled in tertiary educational institutions in Thailand between July 2022 and October 2022. Twenty-four universities across Thailand were invited to participate in the study, of which fourteen agreed to take part (58.3% response rate). The participating universities were located across Thailand, with ten universities in the central region, two in the northern region and one each in the northeastern and southern regions. The list of the participating universities is available in Supplementary Material Figure S1.

Eligible participants were international students (aged ≥18 years), whose primary intention for their trip was to study in Thailand. Those who previously stayed in Thailand as expatriates, and students who had been staying in Thailand for more than 1 year, were excluded from the study.

### 2.2. Recruitment and Data Collection Methods

Following approval by the Ethical Committee of the Faculty of Tropical Medicine at Mahidol University, Thailand (IRB#: MUTM 2022-042-01, 21 June 2022), information about the study was distributed to staff members from participating universities. Recruitment of participants was carried out using convenience sampling, with invitations sent via email and advertisements through social networking platforms such as LINE and Facebook. Interested participants accessed the study’s questionnaire through the Uniform Resource Locators (URL) link or quick response (QR) code attached in the e-mail and advertisements.

### 2.3. Materials

The self-administered questionnaire was developed using the web-based online form builder Jotform^®^ (https://www.jotform.com, accessed on 20 February 2022). Participants were provided with a participant information sheet and consent form before taking part in the survey. An anonymous survey platform was utilised to maintain confidentiality of all participants. The questionnaire consisted of five sections. Firstly, participants were asked to provide their demographic data, including age, sex, nationality, education profile, previous travel in Southeast Asia and history of pre-existing health conditions. The second section assessed participants’ pre-travel preparation, including reasons for seeking or not seeking professional advice, sources of professional and non-professional advice and their immunisation history. The third section comprised six questions, assessing participants’ knowledge of six different travel health topics, namely mosquito-borne diseases, rabies prevention, sexually transmitted diseases, management of travellers’ diarrhoea, awareness of Thailand’s emergency services number and Thai traffic rules. The participants were required to select all the correct answers to each question. The fourth section examined adherence to preventive measures such as food safety, insect repellent usage, condom usage and helmet usage (see Appendix A for further details). The fifth section explored travel-related illnesses that participants may have experienced during their trip, including travellers’ diarrhoea, acute febrile illness, respiratory illness, skin diseases, animal contacts and traffic injuries. Some questions in Section 1, Section 2, Section 3, Section 4 and Section 5 had textboxes for participants to provide additional details. All written responses were evaluated quantitatively. A pilot survey was conducted on an independent group of 14 students to assess the validity of the questionnaire. Minor modifications to the wording were made following the test. Details regarding the presentation of the questionnaire are provided in Supplementary Materials Figure S2.

### 2.4. Sample Size Calculation

Based on the data from the Thailand Office of Higher Education Commission, there were a minimum of 20,000 international students annually attending full-time programs in Thailand’s tertiary education institutions over the past 5 years [6]. Assuming an overall uptake of professional pre-travel advice among international university students of 30% [13,17], and setting a confidence interval of 95% (95% CI), the required sample size was estimated to be 318 using OpenEpi^®^ software version 2.0.

### 2.5. Statistical Analysis

The data collected from the survey were exported from Jotform^®^ database system to SPSS software version 26.0 for data analysis. Continuous data were reported as means with standard deviations (SD) for normally distributed data, while medians with interquartile ranges (IQR) were presented for non-normally distributed continuous data. Categorical data were presented as numbers (*n*) and percentages (%). Chi-Square (Χ^2^) test was used to compare the categorical data between the two groups. Inferential statistics were performed to determine the likelihood of receiving professional pre-travel advice. The variables with a *p*-value less than 0.10 in the Chi-Square analysis were then subjected to univariate logistic regression to calculate crude odds ratios (OR). The variables identified in the univariate analysis were included in multiple logistic regression models to determine independent factors associated with the uptake of professional pre-travel health advice. Adjusted odds ratios (aOR) with the 95% CI were reported for each variable. A *p*-value of less than 0.05 was considered statistically significant.

## 3. Results

### 3.1. Demographic Characteristics

Between July 2022 and October 2022, 324 international students completed the questionnaire. Table 1 displays the demographic characteristics of eligible participants. The gender distribution was almost equally divided, with 50.3% male and 49.7% female. The median age was 24 years, with an interquartile range (IQR) of 21–48 years. The majority of the students originally resided in Asia and Oceania (79.0%; *n* = 256), followed by Europe (16.4%; *n* = 53), Africa (3.4%; *n* = 11) and North and South America (1.2%; *n* = 4). The top five inbound countries were Myanmar (*n* = 119), Indonesia (*n* = 28), Cambodia (*n* = 18), Nepal (*n* = 18) and France (*n* = 16). Most students were enrolled in full-time programs, with the highest number studying for master’s degrees (37.7%; *n* = 122), followed by bachelor’s degrees (28.4%; *n* = 92) and doctoral degrees (15.7%; *n* = 51). Healthcare students comprised 22.9% of the cohort. The median duration of stay since the first arrival was 2.5 months (IQR; 1.6–3.8). The median expected total duration of stay was 24.3 months (IQR; 13.0–52.1). Nearly half of the respondents (49.1%) had previously visited Southeast Asia. Only 17 (5.2%) students reported having pre-existing comorbidities, with an allergy being the most common condition (*n* = 5).

### 3.2. Characteristics of Pre-Travel Preparation

#### 3.2.1. Demographic Characteristics between the Two Groups

Of 324 international students, 174 (53.7%) received at least one source of professional pre-travel advice before visiting Thailand. Table 1 illustrates the differences in demographic characteristics between students who received professional advice and those who did not. The analysis revealed that female students had a higher likelihood to seek professional pre-travel advice than their male counterparts (58.4% vs. 49.1%). European students had the highest uptake rate (81.1%), while Asian students had the lowest (47.7%). Students pursuing bachelor’s degrees showed the lowest proportion of receiving professional advice (38.0%) compared to students in other programs, where the uptake rate was over 50.0%.

#### 3.2.2. Factors Associated with Seeking Professional Pre-Travel Advice

Table 2 presents the likelihood of international students receiving professional pre-travel advice. In the initial analysis using the Chi-Square (X^2^) test, four baseline characteristics had a *p*-value of < 0.10: gender, region of residence, academic programs in Thailand and expected total duration of stay. After performing a multivariate logistic regression analysis, the result indicated that female students were 1.7 times more likely to receive professional pre-travel advice than male students (aOR: 1.73, 95% CI: 1.07–2.81). European students sought professional pre-travel advice 5.6 times more frequently than students from Asia and Oceania (aOR: 5.651, 95% CI: 1.93–16.51). In addition, students enrolled in master’s degrees were almost four times more likely to seek professional pre-travel advice than bachelor’s students (aOR: 3.94, 95% CI: 1.47–10.50). Similarly, those studying for doctoral degrees had a 2.6 times higher tendency to receive professional pre-travel advice than bachelor’s students (aOR: 2.68, 95% CI: 1.26–5.69).

#### 3.2.3. Reasons for Receiving Professional Pre-Travel Advice

**Figure 1:**
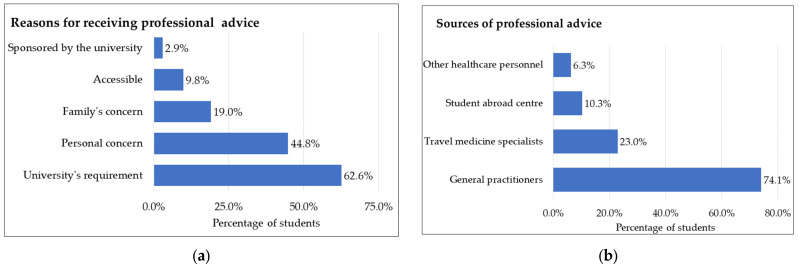
Characteristics of professional advice. (**a**) Reasons for receiving professional advice; (**b**) sources of professional advice. Figure calculated as *n*/number of the students receiving professional advice (*n* = 174).

Figure 1a illustrates the reasons for seeking professional pre-travel advice among international students. Most international students (62.6%) attended pre-travel consultation to comply with the host university’s health examination and vaccination prerequisites, while personal health concerns were the second most common reason (44.8%). Nearly one-fifth of the students sought professional pre-travel advice due to their family’s concern for the students’ health (19.0%). Only 9.8% of students sought professional advice because of its accessibility, while 2.9% accessed advice from sponsored agencies.

#### 3.2.4. Sources of Professional Advice

The primary source of advice was from general practitioners, accounting for 74.1% of respondents. Travel medicine specialists were also a popular choice among students, accounting for 23.0%, followed by staff of study abroad centres at 10.3% and other healthcare personnel such as nurses at 6.3%. These findings are presented in Figure 1b.

#### 3.2.5. Reasons for Not Receiving Professional Pre-Travel Advice

**Figure 2:**
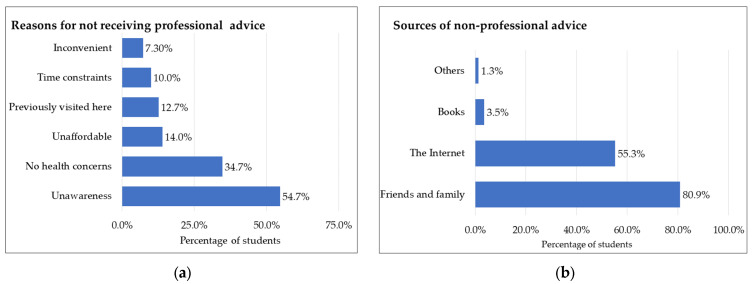
Barriers to professional advice and other sources of pre-travel advice. (**a**) Reasons for not receiving professional advice. Figure calculated as *n*/number of students not receiving professional advice (*n* = 150); (**b**) sources of non-professional advice. Figure calculated as *n*/number of the students responding to the question (*n* = 226).

Out of 324 international students, 150 did not receive professional pre-travel advice. Over half of them (54.7%) did not seek advice because they were unaware of the service. One-third (34.7%) did not have health concerns for their trip. Affordability was also a factor for 14% of respondents. Other cited barriers included having visited the Southeast Asian region previously (12.7%) and time constraints (10%). A small percentage (7.3%) found the consultation inconvenient (Figure 2a).

#### 3.2.6. Sources of Non-Professional Advice

Out of 226 respondents, 80.9% received pre-travel advice from family and friends, while 55.3% relied on the Internet. Merely 3.5% of respondents reported using books as a source of information, and 1.3% obtained advice from other sources such as university briefings, insurance companies and travel agencies (Figure 2b).

#### 3.2.7. Immunisation History

Among 324 international students, the COVID-19 vaccine was obtained by almost all respondents (96.0%) prior to their visit to Thailand, followed by the hepatitis B vaccine (44.4%). The uptake rate of the measles-containing vaccine was reported at 31.2%, while influenza and hepatitis A vaccines had a similar uptake rate of 29.6%. The tetanus-containing vaccine was reported by 26.2% of all students. Over 17.9% received the rabies vaccine, followed by human papillomavirus (16.3%), dengue (16.0%) and Japanese encephalitis (15.7%), respectively. Individuals who sought professional pre-travel advice exhibited higher rates of uptake across all vaccines compared to their counterparts. A statistical analysis of 10 vaccines indicated that students who sought professional advice had a statistically significant higher uptake rate for 6 vaccines, namely, hepatitis A, a booster dose of the tetanus-containing vaccine, measles-containing virus, human papillomavirus, rabies and Japanese encephalitis (Table 3).

### 3.3. Knowledge of Travel-Related Health Problems

In this section, six multiple-answer questions were used to assess knowledge of travel-related health issues. Only 31.8% of the students correctly identified Japanese encephalitis (JE) as a mosquito-borne disease. However, those who received professional advice had a higher likelihood of recognising JE than those who did not (*p*-value: 0.011). In terms of rabies prevention, less than a quarter of respondents (22.5%) recognised the importance of receiving a tetanus booster dose along with the rabies vaccine, and less than half (43.5%) were aware of the need for an adequate wound cleansing. Moreover, the knowledge of sexually transmitted diseases (STDs) was low, as only 38.6% of respondents identified hepatitis B as an STD, and only 26.9% knew that hepatitis A can also be transmitted through sexual contact. The question on management of travellers’ diarrhoea found that 26.9% of respondents incorrectly believed that antibiotics are the mainstay of treatment for the condition and should be taken regardless of symptom severity. In addition, students had suboptimal knowledge of non-infectious health risks, as only 41.7% correctly identified Thailand’s emergency services number as 191, and more than one-fifth of the students (21.5%) mistakenly believed that vehicles in Thailand drive on the right side of the road (Table 4).

### 3.4. Preventive Practices

#### 3.4.1. Food to Consume with Caution

**Figure 3:**
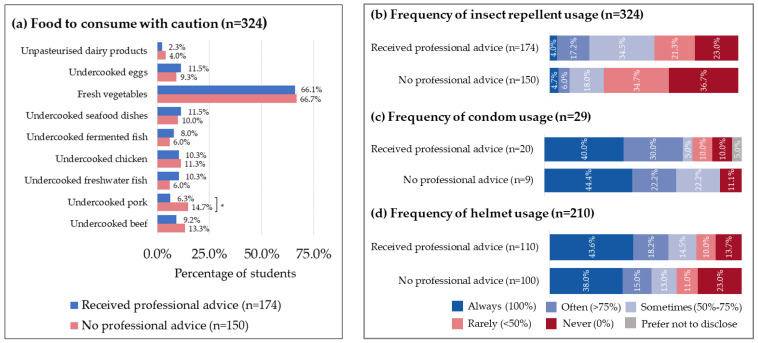
Preventive practices. (**a**) Food to consume with caution. Figure calculated as *n*/number of students receiving professional advice (*n* = 174) and *n*/students not receiving professional advice (*n* = 150). * *p* < 0.05. (**b**) Frequency of insect repellent use. (**c**) Frequency of condom usage of those with new casual sex partners (*n* = 29). (**d**) Frequency of helmet usage of those with history of riding a motorcycle (*n* = 210).

There was no significant difference in the proportion of students consuming all the listed foods between those who sought professional advice and those who did not, except for undercooked pork. The consumption rate of other foods on the list was similar between the two groups. The percentage of students not seeking professional advice who consumed undercooked pork was significantly higher than those who sought advice (14.7% vs. 6.3%, *p*-value: 0.013). Both groups reported consuming fresh vegetables at a rate of over 60% (66.1% vs. 66.7%) (Figure 3a).

#### 3.4.2. Insect Repellent Usage

The usage of insect repellent was almost the same in both the group that received professional advice and the group that did not, with approximately 4% of the students reporting “always” using it (Figure 3b). However, the group seeking professional advice had a higher percentage of students reporting “often” using insect repellent compared to the other group (17.2% vs. 6.0%). Similarly, a higher proportion of students seeking professional advice reported “sometimes” using insect repellent than those not seeking advice (34.5% vs. 18.0%). Conversely, more students not seeking professional advice reported “rarely” using insect repellent and “never” using insect repellent compared to those seeking professional advice (34.7% vs. 21.3% and 36.7% vs. 23.0%, respectively).

#### 3.4.3. Condom Usage

Of the 29 respondents who reported having new casual sex partners while in Thailand, 20 students received professional pre-travel advice, while 9 were in the other group. Of those who “always” wore condoms, 8 received professional advice. A higher proportion of those who received professional advice reported “often” using condoms compared to the other group. The proportion of those who reported “sometimes”, “rarely” or “never” using condoms was similar between the two groups (Figure 3c).

#### 3.4.4. Helmet Usage

Out of 210 students who had ridden a motorcycle, approximately two-thirds of both groups received or did not receive professional advice (*n* = 110; 63.2% vs. *n* = 100; 66.7%). Less than half of the students in each group reported “always” wearing a helmet (43.6% vs. 38.0%). Students not receiving professional advice (Figure 3d) were more likely to report “never” wearing a helmet compared to the other group (23.0% vs. 13.7%).

### 3.5. Travel-Related Illnesses

#### 3.5.1. Reports of Travel-Related Illnesses

Among 324 international students surveyed, 31.5% (*n* = 102) reported acquiring one or more travel-related illnesses during their stay in Thailand. The most reported illnesses were respiratory illnesses (18.2%), followed by acute febrile illness (15.4%) and travellers’ diarrhoea (14.5%). Dermatologic diseases and animal contacts were reported by 13.6% and 3.0% of students, respectively. Thirteen students (4.0%) reported other illnesses, including mental health issues (two with depression and one with anxiety), eye infections, insomnia, headache, jet lag, urinary stone, gout, abdominal pain, an allergic reaction to an insect and dysmenorrhoea (Table 5).

#### 3.5.2. Health-Seeking Behaviours

Of all illness episodes experienced by the surveyed international students in Thailand, more than half were self-treated (58.3%), while one-fifth resolved spontaneously (19.7%). Around 17.0% of illnesses required professional treatment in out-patient departments, and 5.0% needed hospitalisation. Most illnesses were self-treated except for the “others” group, where 46.3% sought professional care. The group “acute febrile illness” had the highest number of hospitalised cases (*n* = 5), followed by respiratory (*n* = 3), others (*n* = 2) and dermatologic (*n* = 1). All cases of animal contact involved mammals, specifically cats (*n* = 8) and dogs (*n* = 2). However, only 2 of the 10 individuals received rabies post-exposure prophylaxis, while the rest treated the injuries themselves (Table 5).

## 4. Discussion

To the best of our knowledge, this is the first study to explore the travel health behaviours among culturally diverse international students in Southeast Asia, encompassing various aspects of travel health preparedness. Despite consisting mostly of Asian students, approximately half of the students received professional pre-travel advice prior to their trip to Thailand, which is an encouraging finding compared to the previous literature, which reported rates as low as 0% to 25% among Asian students [15,16,17]. However, our analysis indicates that seeking professional pre-travel advice is still more prevalent among Western students than Asian students, a pattern that has been previously reported in previous research involving domestic and international students attending an Australian university [8]. This may be attributed to the fact that travel medicine has been developed and cultivated in the Western world for several decades, whereas it is a relatively new practice in Asia [18,21]. As a result, many Asian students may not be accustomed to pre-travel preparation unless required by their host university to undergo certain health examinations and vaccinations, as our study’s findings also corroborate. The majority of students in our study sought professional advice primarily due to the university’s requirement for mandatory health examination and vaccination.

The primary providers of pre-travel health services for our study cohort were general practitioners, which is consistent with previous research [9,11,12]. This may be due to their widespread availability and ability to offer comprehensive management during consultations, regardless of location. In countries with limited availability of travel medicine specialists, general practitioners may be the only viable option for pre-travel health services. However, previous studies have noted that some primary care providers in Asian countries are not familiar with travel medicine, leading to limited access to quality pre-travel health preparation [21]. For instance, in our study, we found that despite Myanmar being the leading inbound country, there were no facilities providing professional travel health services for Burmese students based on the information listed on the official webpage of the International Society of Travel Medicine’s global travel clinic directory (https://www.istm.org/AF_CstmClinicDirectory.asp, accessed on 20 February 2022). This lack of visibility and accessibility may explain why half of the respondents did not seek advice. It is important to note that while numerous general practitioners offer pre-travel consultations, they may not have undergone professional training in travel medicine, potentially leading to discrepancies in information and management compared to certified travel medicine specialists. Therefore, it is crucial to expand professional travel medicine training to healthcare practitioners in non-Western countries and to educate young adult travellers in these regions on the importance of pre-travel preparation.

A notable proportion of students in the study did not seek professional pre-travel advice due to the absence of perceived health concerns for their trip. This complacency towards travel-related health risks may be attributed to the belief that they were healthy and had completed all necessary vaccinations during childhood [8,18]. The familiarity of the destination may also have played a role in the decision to forego professional advice, as half of the respondents had previously been to Southeast Asia. This finding is in line with the results of a study by Sohail A et al. (2022) [18], which found that previous travel experiences without illness were linked to the perception that pre-travel preparation was unnecessary. Furthermore, as most international students in the survey were from Asian countries, they may perceive travel to Thailand as a low-risk activity due to its proximity and cultural similarities. This pattern of behaviour has been observed in a study of Japanese students, which found that those travelling to East Asian countries obtained health information less frequently than those travelling to other destinations [16]. This finding highlights the need for travel medicine practitioners to educate travellers that even travelling to nearby countries can pose potential health and security risks. For instance, the seroprevalence of viral hepatitis A in Thailand is notably lower compared to its neighbouring countries [22]. Additionally, it is crucial to note that the safety and security levels of neighbouring countries’ borders can significantly differ from one another [23].

Family and friends and the Internet were the most popular sources of non-professional pre-travel health advice, consistent with previous studies [8,12,15]. Seeking advice from family and friends may be popular due to their personal experiences and opinions, which are more relatable and easier to understand than professional advice. Prior research has shown that students often turn to the Internet for travel health advice due to its accessibility and up-to-date nature, as well as the absence of consultation fees [18]. The emergence of ChatGPT, an AI-powered chatbot, is expected to become the next generation’s primary source of non-professional pre-travel advice, offering basic guidance based on user input. However, the advice given by this platform is general and not personalised, and relying exclusively on it may not be optimal. Hence, there is a need to create a reliable online resource of pre-travel information tailored to the needs of young adults.

Vaccination uptake rates for all vaccines, except COVID-19, were found to be below 50%. However, the study’s reliance on the respondents’ recall memory may have limited the accuracy of these findings. Nonetheless, the research revealed that students who received professional pre-travel advice had significantly higher vaccination uptake rates for several vaccines. Seeking professional advice before travelling may be a good opportunity for individuals to catch up on missed childhood and travel vaccinations. This can be particularly important for preventing the spread of infectious diseases that have been known to cause outbreaks in university campuses, such as mumps and measles [24,25,26]. Considering the limited access to quality pre-travel counselling for international students from resource-limited countries, host universities can implement an on-campus vaccination campaign to catch up on missed vaccinations, as has proven effective in previous studies [27,28].

The students’ level of travel health knowledge was suboptimal, with a significant number of students not aware of Japanese encephalitis despite residing in endemic areas. However, those who received professional advice were more likely to be aware of the disease, indicating the importance of seeking pre-travel advice from a travel medicine specialist [29]. Despite receiving professional advice, many students still neglected important rabies preventive measures and had misconceptions about rational antibiotic use for travellers’ diarrhoea. Moreover, many lacked general knowledge about the countries they were visiting, including traffic rules and emergency service numbers. These findings suggest that traditional travel medicine practice may not be sufficient, and efforts should be made to emphasise non-infectious health risks as well. Therefore, healthcare providers responsible for pre-travel preparation among international students must discuss common travel-related health problems during consultations, in addition to mandatory health examinations and vaccines. To make the information more digestible, collaborating with digital platforms that provide engaging educational materials could be a potential solution.

The study found that more than half of the respondents reported consuming “fresh vegetables”, which was an error in the questionnaire as the intention was to assess the consumption of “raw vegetables”. Although the interpretation of this result may be questionable, previous research has shown that consuming raw vegetables is associated with the occurrence of travellers’ diarrhoea in Southeast Asia [30,31]. It is therefore important to advise travellers to be cautious when consuming raw vegetables as they are often included in Asian dishes. The present study found that individuals who did not seek pre-travel preparation reported consuming a significantly higher amount of uncooked pork compared to those who sought pre-travel advice, indicating a potential association between seeking professional pre-travel advice and preventive practices against travellers’ diarrhoea. However, more research is necessary to identify factors hindering adherence to food precautions among international students.

Participants who sought pre-travel counselling were more likely to use insect repellent regularly than those who did not. This finding is consistent with a previous survey that demonstrated a significantly higher adherence to preventive measures against mosquito-borne diseases among those who received professional pre-travel advice [32], suggesting that access to professional pre-travel resources can have a significant impact on the preventive practices of students.

Condom use was low among students with new casual sex partners, consistent with prior research on international students [33,34]. Possible reasons for this include unplanned sexual activity, alcohol consumption, preference for condomless sex, stigma associated with condom use [34] and low perceived risk of sexually transmitted infections [8]. Given the prevalence of sexually transmitted infections in host countries [35], promoting safe sex practices during pre-travel counselling is paramount.

Poor adherence to road safety was observed in this study and has been noted in previous research on Swedish students travelling abroad [13]. The local practices in the areas where the students attended university, where non-helmet use was more prevalent, may have influenced their behaviour [36]. The students’ reluctance to use helmets provided by motorcycle taxis due to unsanitary conditions may also contribute to the poor compliance. To promote personal safety, pre-travel counselling should include road safety practices, in addition to infectious disease risks [37]. Future research should focus on helmet use among international travellers, as prior surveys have focused more on seatbelt use [13,38].

A third of the students reported experiencing travel-related illnesses in this survey, which aligns with previous studies conducted among international students and general travellers visiting Southeast Asia [12,13,39]. Respiratory illnesses, acute febrile illnesses and travellers’ diarrhoea were the most prevalent illnesses reported in this study, consistent with previous surveys among this traveller group [13,17,19]. None reported traffic-related injuries in this study. This finding contradicts previous research among international students, where the incidence of traffic-related injuries ranged from 0.8% to 8.0% [12,13,14]. The reasons for this unexpected result are unclear, but it is possible that the COVID-19 pandemic’s travel limitations may have contributed to the outcome. Many students may have chosen to stay on campus, reducing their travel and transportation activities and thereby decreasing the probability of traffic-related injuries. Future research conducted after the lifting of travel restrictions may provide more representative results. Three out of thirteen students reported having mental health conditions (two with depression and one with anxiety), which is significantly lower than previous studies among Western students [12,14]. This may be due to the familiarity with sociocultural factors among predominantly Asian students in the survey who had been to Southeast Asia before, while students from other continents may struggle more to adjust to a new environment, leading to higher rates of mental health problems [40].

Students generally preferred to self-treat their health problems. Possible reasons for this include students perceiving their health problems as mild and relying on non-professional sources for treatment information. Some may also refrain from seeking professional care due to difficulties in accessing healthcare facilities or a lack of trust in the quality of care in the host country [13]. Financial constraints may also hinder seeking professional care in some cases. However, not seeking professional healthcare may exacerbate some illnesses, underscoring the importance of further research into identifying barriers that prevent this population from seeking local healthcare facilities.

### Limitations and Future Research

Cross-sectional self-report questionnaires can be prone to reliability issues, including recall bias, social desirability bias and confounding. For example, respondents were asked about their past immunisation history and travel-related illnesses, which may be subject to inaccuracies due to the difficulty in recalling such information. Additionally, sexual health history may be a sensitive subject for some participants, leading to potential inaccuracies in responses from certain regions. To mitigate recall biases, future studies should use documented histories or prospective designs. Qualitative approaches with confidentiality should be used for research on sensitive subjects to ensure participant privacy. The present study was limited by time constraints and only collected data from a single survey of the student population. This approach may not provide a complete view of travellers’ experiences, as some respondents had not yet been exposed to travel-related health risks. A follow-up survey in the coming weeks, focusing on the participants’ preventative practices after exposure to certain health risks and travel-related illnesses, could provide a more comprehensive understanding of this cohort’s experiences.

## 5. Conclusions

Despite high uptake of professional pre-travel advice prior to visiting Thailand, this study identified insufficient knowledge and suboptimal preventive practices regarding travel health among international students. On-campus pre-travel assessments and immunisation programs could enhance the quality of pre-travel consultations for students who lack access to professional pre-travel sources in their home countries. Furthermore, raising awareness of pre-travel preparedness through targeted campaigns delivered via popular platforms for young adults may encourage students to seek professional pre-travel preparation, increasing accessibility to travel medicine practices globally. Considering this, travel medicine can function as a public health tool to improve overall health outcomes beyond individuals.

## Figures and Tables

**Table 1:** Demographic characteristics of total participants (*n* = 324), those who received professional advice (*n* = 174) and those who did not (*n* = 150).

Variables	Total *n* = 324		Received Professional Advice *n* = 174 (53.7%)		Did Not Receive Professional Advice *n* = 150 (46.3%)		*p*-Value (X^2^)
	*n*	%	*n*	%	*n*	%	
Gender							
Female	163	50.3	94	58.4	67	41.6	0.093 ^c^
Male	161	49.7	80	49.1	83	50.9	
Age, years (Median = 24, IQR = 21–48)							
18–20	146	45.1	84	57.5	62	42.5	0.267
21–25	60	18.5	29	48.3	31	51.7	
25–30	58	17.9	34	58.6	24	41.4	
>30	60	18.5	27	45.0	33	55.0	
Region of residence ^$^							
Asia and Oceania	256	79.0	122	47.7	134	52.3	<0.001 ^c^
Europe	53	16.4	43	81.1	10	18.9	
Africa	11	3.4	7	63.6	4	36.4	
North and South America	4	1.2	2	50.0	2	50.0	
Academic program in Thailand							
Bachelor’s degree	92	28.4	35	38.0	57	62.0	0.006 ^c^
Master’s degree	122	37.7	69	56.6	53	43.4	
Doctoral degree	51	15.7	29	56.9	22	43.1	
Semester abroad	38	11.7	28	73.7	10	26.3	
Short program ^†^	12	3.7	7	58.3	5	41.7	
Elective or visiting programs	9	2.8	6	66.7	3	33.3	
Field of Study							
Healthcare	71	22.9	35	49.3	36	50.7	0.399
Non-healthcare	253	77.1	139	54.9	114	45.1	
Duration of stay since arrival, months (Median = 2.4, IQR = 1.6–3.8)							
<1 month	40	12.3	23	57.5	17	42.5	0.357
1–2 months	97	29.9	51	52.6	46	47.4	
2–3 months	77	23.8	46	59.7	31	40.3	
3–4 months	42	13.0	17	40.5	25	59.5	
>4 months	68	21.0	37	54.4	31	45.6	
Expected total duration of stay, months (Median = 26.1, IQR = 13.0–52.1)							
<6 months	58	17.9	36	67.2	19	32.8	0.012 ^c^
6–12 months	28	8.6	20	71.4	8	28.6	
12–24 months	89	27.5	45	50.6	44	49.4	
>24 months	149	46.0	60	47.0	79	53.0	
Previous visit to Southeast Asia							
Yes	159	49.1	84	52.8	75	47.2	0.757
No	165	50.9	90	54.5	75	45.5	
Pre-existing comorbidities							
Yes ^‡^	17	5.2	11	64.7	6	35.3	0.350
No	307	94.8	163	53.1	144	46.9	

Notes: ^$^ Top 5 inbound countries: Myanmar (*n* = 119), Indonesia (*n* = 28), Cambodia (*n* = 18), Nepal (*n* = 18) and France (*n* = 16). ^†^ Diploma (*n* = 9) and Certificates (*n* = 3). ^‡^ Allergy (*n* = 5), mental health conditions (*n* = 3), cardiovascular (*n* = 2), respiratory (*n* = 2), diabetes mellitus (*n* = 1), gastrointestinal (*n* = 1), immunocompromised (*n* = 1) and others (*n* = 4); left transhumeral amputation, vitamin D deficiency, rheumatoid arthritis and hearing impairment (each participant could choose more than 1 comorbidity). ^c^ *p* < 0.10.

**Table 2:** Factors associated with seeking professional pre-travel advice among international students.

Variables	Univariate ^a^			Multivariate ^a^		
	OR	95% CI		aOR	95% CI	
		Lower	Upper		Lower	Upper
Gender						
Male ^RV^	1.00	-	-	1.00	-	-
Female	1.46 *	0.94	2.26	1.73 *	1.07	2.81
Region of residence						
Asia and Oceania ^RV^	1	-	-	1	-	-
Europe	4.72 ***	2.27	9.80	5.65 **	1.93	16.51
Africa	1.92 ^ns^	0.55	6.73	2.40 ^ns^	0.64	8.89
North and South America	1.10 ^ns^	0.15	7.92	0.99 ^ns^	0.11	8.71
Academic program in Thailand						
Bachelor’s degree ^RV^	1	-	-	1	-	-
Master’s degree	2.12 **	1.22	3.68	3.94 **	1.47	10.50
Doctoral degree	2.15 *	1.07	4.30	2.68 *	1.26	5.69
Semester abroad	4.56 ***	1.98	10.52	2.76 ^ns^	0.46	18.18
Short program ^†^	2.28 ^ns^	0.67	7.74	0.86 ^ns^	0.11	6.68
Elective or visiting programs	3.26 ^ns^	0.76	13.86	2.11 ^ns^	0.25	17.80
Expected total duration of stay (months)						
<6 months RV	1	-	-	1	-	-
6–12 months	1.22 ^ns^	0.45	3.26	1.44 ^ns^	0.28	7.21
12–24 months	0.50 ^ns^	0.25	0.99	0.74 ^ns^	0.16	3.46
>24 months	0.43 ^ns^	0.23	0.81	1.56 ^ns^	0.34	7.21

Abbreviations: OR, odds ratio; CI, confidence interval; ^RV^ reference value; ^a^ *p*-value threshold: *** *p* < 0.001, ** *p* < 0.01, * *p* < 0.05, ^ns^ not significant.

**Table 3:** Immunisation history of total participants (*n* = 324), those who received professional advice (*n* = 174) and those who did not (*n* = 150).

Vaccine	Total *n* = 324		Received Professional Advice *n* = 174 (53.7%)		Did Not Receive Professional Advice *n* = 150 (46.3%)		*p*-Value (X^2^)
	*n*	%	*n*	%	*n*	%	
COVID-19	311	96.0	166	95.4	145	96.7	0.563
Hepatitis B	143	44.1	87	50.0	56	37.3	0.054
Measles-containing vaccine	101	31.2	67	38.5	34	22.7	0.007 *
Hepatitis A	96	29.6	69	39.6	27	18.0	<0.001 *
Influenza	96	29.6	56	32.2	40	26.7	0.187
Tetanus-containing vaccine	85	26.2	59	33.9	26	17.3	0.002 *
Rabies	58	17.9	41	23.5	17	11.3	0.013 *
Human papillomavirus	53	16.3	39	22.4	14	9.3	0.006 *
Dengue	52	16.0	29	16.7	23	15.3	0.947
Japanese encephalitis	51	15.7	37	21.3	14	9.3	0.013 *

* *p* < 0.05

**Table 4:** Knowledge of travel-related health problems.

Knowledge of Travel-Related Health Problems	Total *n* = 324		Received Professional Advice *n* = 174 (53.7%)		Did Not Receive Professional Advice *n* = 150 (46.3%)		*p*-Value (X^2^)
	*n*	%	*n*	%	*n*	%	
**Mosquito-borne diseases**							
“Recognised JE as a mosquito-borne disease”	103	31.8	66	37.9	37	24.7	0.011 *
**Rabies prevention**							
“Administer the tetanus vaccine along with rabies vaccine if the previous was more than 10 years”	73	22.5	36	20.7	37	24.7	0.393
“Acknowledged the importance of adequate wound cleansing post animal bites”	141	43.5	84	48.3	57	38.0	0.063
**Sexually transmitted diseases**							
“Recognised hepatitis B as an STD”	125	38.6	60	34.5	65	43.3	0.103
“Recognised hepatitis A as an STD”	87	26.9	43	24.7	44	29.3	0.349
**Travellers’ diarrhoea management**							
“Misunderstood that antibiotics should be taken immediately regardless of the symptom’s severity.”	87	26.9	44	25.3	43	28.7	0.494
**Thailand’s emergency services number**							
“Recognised that 191 is Thailand’s emergency services number”	135	41.7	75	43.1	60	40.0	0.572
**Thailand’s traffic rules**							
“Misunderstood that Thailand’s vehicles drive on the right side of the road”	70	21.6	34	19.5	36	24.0	0.331

Abbreviations: JE, Japanese encephalitis; STD, sexually transmitted disease. * *p* < 0.05

**Table 5:** Reports of travel-related illnesses and the students’ health-seeking behaviours.

Travel-Related Illness	Students with Any Reported Illnesses, 102/324 (31.5%)									
	Reported Illnesses		Health-Seeking Behaviour for Each Illness							
			Spontaneously Resolved		Self-Treated		Out-Patient Care		Hospitalised	
	*n*	% ^§^	*n*	*%* ^††^	*n*	% ^††^	*n*	% ^††^	*n*	% ^††^
Respiratory	59	18.2	9	15.3	40	67.8	7	11.9	3	5.1
Acute febrile illness	50	15.4	2	4.0	33	66.0	10	20.0	5	10.0
Travellers’ diarrhoea	47	14.5	16	34.0	25	53.2	6	12.8	-	-
Dermatologic	44	13.6	16	36.4	20	45.5	7	15.9	1	2.3
Animal contacts ^#^	10	3.0	-	-	8	80.0	2 ^b^	20.0	-	-
Traffic injury	-	-	-	-	-	-	-	-	-	-
Others ^¶^	13	4.0	1	7.7	4	30.8	6	46.1	2	15.4
Total	223	-	44	19.7	130	58.3	38	17.0	11	5.0

^§^ Figure calculated as *n*/total participants. ^††^ Figure calculated as *n*/number of illness episodes in that category. ^#^ Contacts with cats (*n* = 8) and contacts with dogs (*n* = 2). ^¶^ Mental health issues (*n* = 3; 2 with depression and 1 with anxiety), eye infection (*n* = 2), insomnia (*n* = 1), jet lag (*n* = 1), urinary stone (*n* = 1), gout (*n* = 1), headache (*n* = 1), abdominal pain (*n* = 1), dysmenorrhoea (*n* = 1) and an allergic reaction to an insect (*n* = 1). ^b^ Received rabies post-exposure prophylaxis.

## Data Availability

Not applicable.

## Supplementary Materials

The following supporting information can be downloaded at: https://www.mdpi.com/article/10.3390/tropicalmed8060322/s1, Figure S1: list of participating universities, Figure S2: presentation of the questionnaire.

## Appendix A

In the fourth section of the questionnaire, compliance with insect repellent usage, condom usage and helmet usage was evaluated by grading the adherence based on five categories: “always” (100%), “often” (>75%), “sometimes” (50–75%), “rarely” (<50%) and “never” (0%). The part on condom usage also included the choice “prefer not to disclose”.
