# Supplementary material for: International University Students’ Pre-Travel Preparation, Knowledge and Practices towards Travel Health in Thailand: A Nationwide Cross-Sectional Study
Source: Trop Med Infect Dis. 2023 Jun 15;8(6):322. doi: 10.3390/tropicalmed8060322 (PMC10303968; doi:10.3390/tropicalmed8060322)
Supplement: Supplementary file 1 [file tropicalmed-08-00322-s001.zip › tropicalmed-2423116-supplementary.pdf]

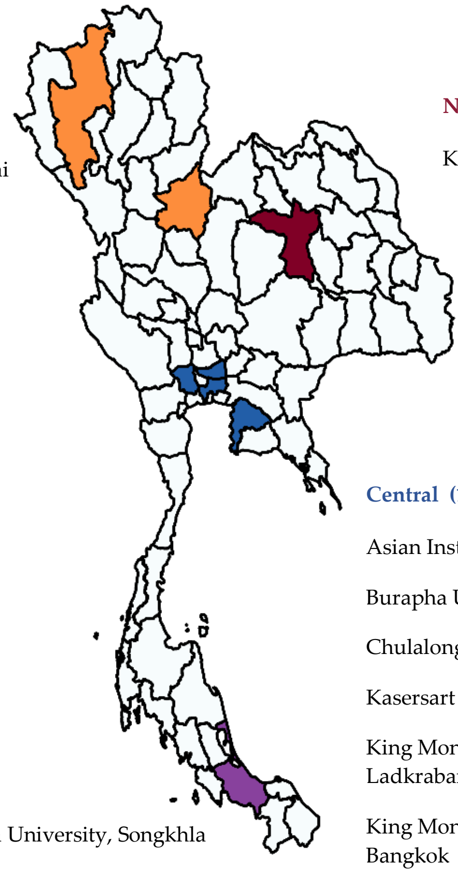

North (2)

Chiang Mai University, Chiang Mai

Naresuan University, Phitsanulok

Northeastern (1)

Khon Kaen University, Khon Kaen

Central (10)

Asian Institute of Technology, Pathum Thani

Burapha University, Chonburi

Chulalongkorn University, Bangkok

Kasersart University, Bangkok

King Mongkutt's Institute of Technology Ladkrabang, Bangkok

King Mongkutt's Institute of Technology Thonburi, Bangkok

Mahidol University, Bangkok

Rajamangala University of Technology Thanyaburi, Pathum Thani

Rangsit University, Pathum Thani

Thammasart University, Bangkok

South (1)

Prince of Songkhla University, Songkhla

Figure S1: List of the participating universities

Pre-travel preparation, travel-related health knowledge and preventive behaviors among international university students in Thailand

Informed Consent

You are invited to participate in a research study about pre-travel health uptake among international university students who enroll in their tertiary education in Thailand. This research study aims to examine therate of pre-travel consultation, level of travel-related health knowledge, and preventive practices. In addition,the study explores the prevalence of common travel-related health illnesses among international university students in Thailand.

This study is being conducted by Sawettachai Jaita,M.D, Faculty of Tropical Medicine, Mahidol University.

There are qualifications to participate in this study:

- International university students.
- Age ≥ 18 years old.
- Primarily traveling to Thailand for educational purposes.
- Able to read and understand the English questionnaire.
- Willing to participate in the study.
- Living in Thailand for less than one year.

Participation in this study is voluntary. If you agree to participate in this study, you would be surveyed by this questionnaire, which will take you no more than 20 minutes. The survey includes 5 sections as follows.

- **Section 1**: Demographic data
- **Section 2**: Pre-travel preparation
- **Section 3**: Knowledge of travel-related health problems
- **Section 4**: Preventive behaviors while staying in Thailand

Participating in this study may not benefit you directly, but the results obtained from this study will provide a piece of background knowledge to develop a novel approach to pre-travel health care in abroad students. The information you will share with us if you participate in this study will be kept confidential to the law's full extent.

If you have any questions about this study, please contact Sawettachai Jaita,M.D via e-mail address: **sawettachai.jai@student.mahidol.edu**

By completing this survey, you are consenting to participate in this study.

Next

Figure S2: Presentation of the questionnaire

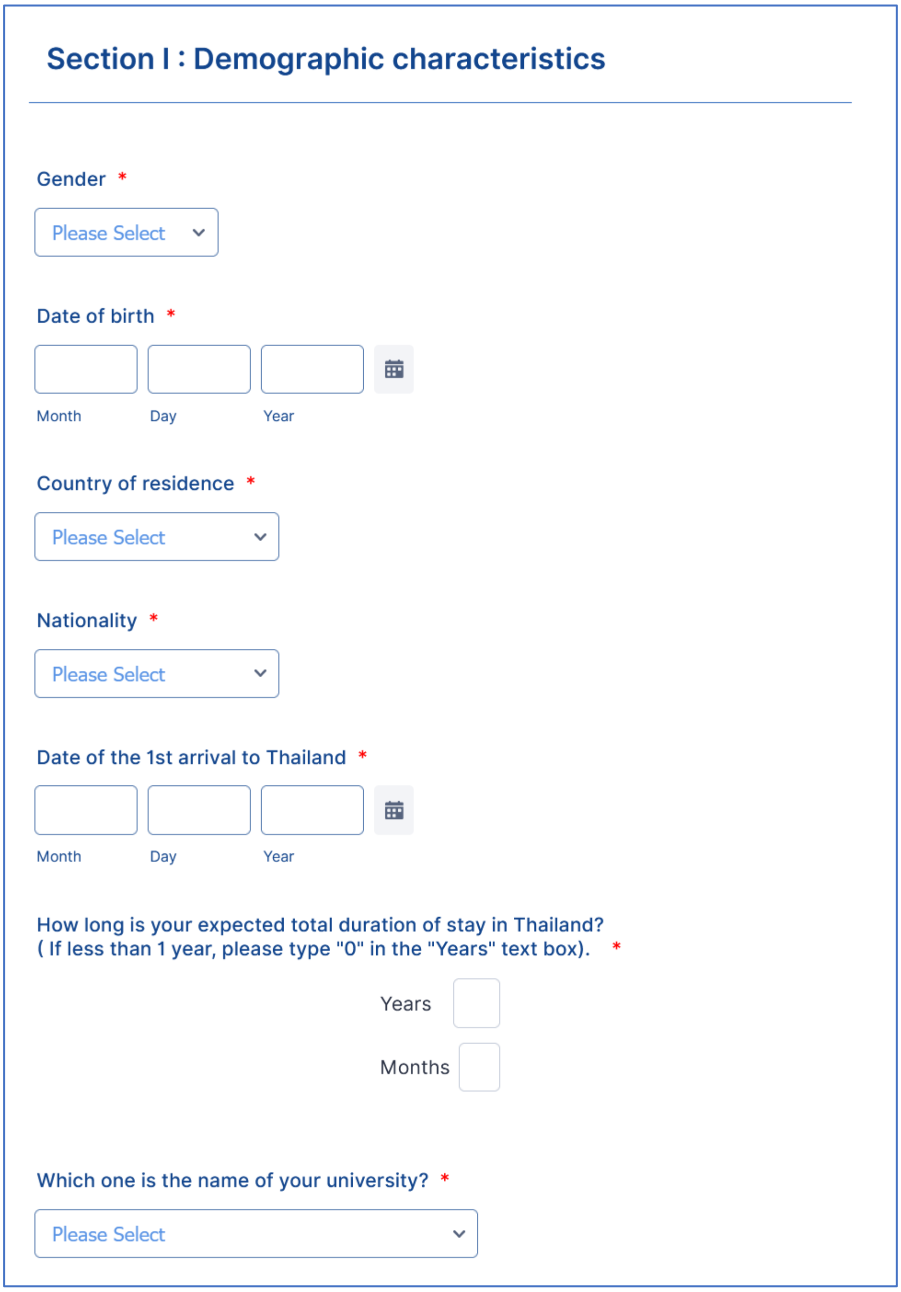

Section I : Demographic characteristics

Gender *

Please Select

Date of birth *

Month | Day | Year

Country of residence *

Please Select

Nationality *

Please Select

Date of the 1st arrival to Thailand *

Month | Day | Year

How long is your expected total duration of stay in Thailand?
(If less than 1 year, please type "0" in the "Years" text box). *

Years

Months

Which one is the name of your university? *

Please Select

Figure S2: Presentation of the questionnaire (cont.)

If answer "Other", please indicate the name of your university. *

What is your current study program? *

What is the type of your study program? *

- [ ] Certificate
- [ ] Diploma
- [ ] Bachelor's degree
- [ ] Master's degree
- [ ] Doctoral degree
- [ ] Short-term exchange program
- [ ] Visiting program
- [ ] Other

Have you ever visited Thailand or other ASEAN countries before? *

- [ ] YES
- [ ] NO

Please select every country that you have visited before this trip. *

- [ ] Brunei
- [ ] Cambodia
- [ ] Laos
- [ ] Indonesia
- [ ] Malaysia
- [ ] Myanmar
- [ ] Philippines
- [ ] SIngpore
- [ ] Thailand (* not including the current visit)
- [ ] Timor leste
- [ ] Vietnam

Do you have any health conditions? *

- [ ] YES
- [ ] NO

Please select the current medical conditions. (*Choose all that apply). *

- [ ] Cardiovascula diseases
- [ ] Diabetes mellitus
- [ ] Dyslipidemia
- [ ] Hypertension
- [ ] Gastrointestinal diseases
- [ ] Immunocompromised
- [ ] Neurological diseases
- [ ] Psychiatric diseases
- [ ] Other

Did you bring any medicines for your medical condition(s) to Thailand? *

- [ ] YES
- [ ] NO

What are the medicines that you brought to Thailand? (*Please provide as much complete information as possible).

Type here...

Figure S2: Presentation of the questionnaire (cont.)

Section 2: Pre-travel preparation

Section 2.1: Source of pre-travel health advice.

Did you seek pre-travel health advice from a medical professional before coming to Thailand? *

- [ ] YES
- [ ] NO

Who did you consult for pre-travel advice?,(Please choose all that apply) *

- [ ] Travel medicine physician
- [ ] General physician / Family physician
- [ ] Nurse
- [ ] University health staff
- [ ] Other

Why did you decide to consult a professional staff for pre-travel advice?,(Please choose all that apply) *

- [ ] Personal concern
- [ ] Family concern
- [ ] Subsidized by the home country's university
- [ ] Vaccinations
- [ ] Accessible
- [ ] Requirement of the university
- [ ] Other

Why did not you seek pre-travel advice from a professional staff?(*Please choose all that apply) *

- [ ] No medical concern
- [ ] Financial constraint
- [ ] Inaccessible
- [ ] Having been to this regions in the past
- [ ] Having had pre-travel advice in the previous trip
- [ ] Other

Did you seek pre-travel information from the internet? *

- [ ] YES
- [ ] NO

Please indicate the name of the website. (*Optional)

Type here...

Figure S2: Presentation of the questionnaire (cont.)

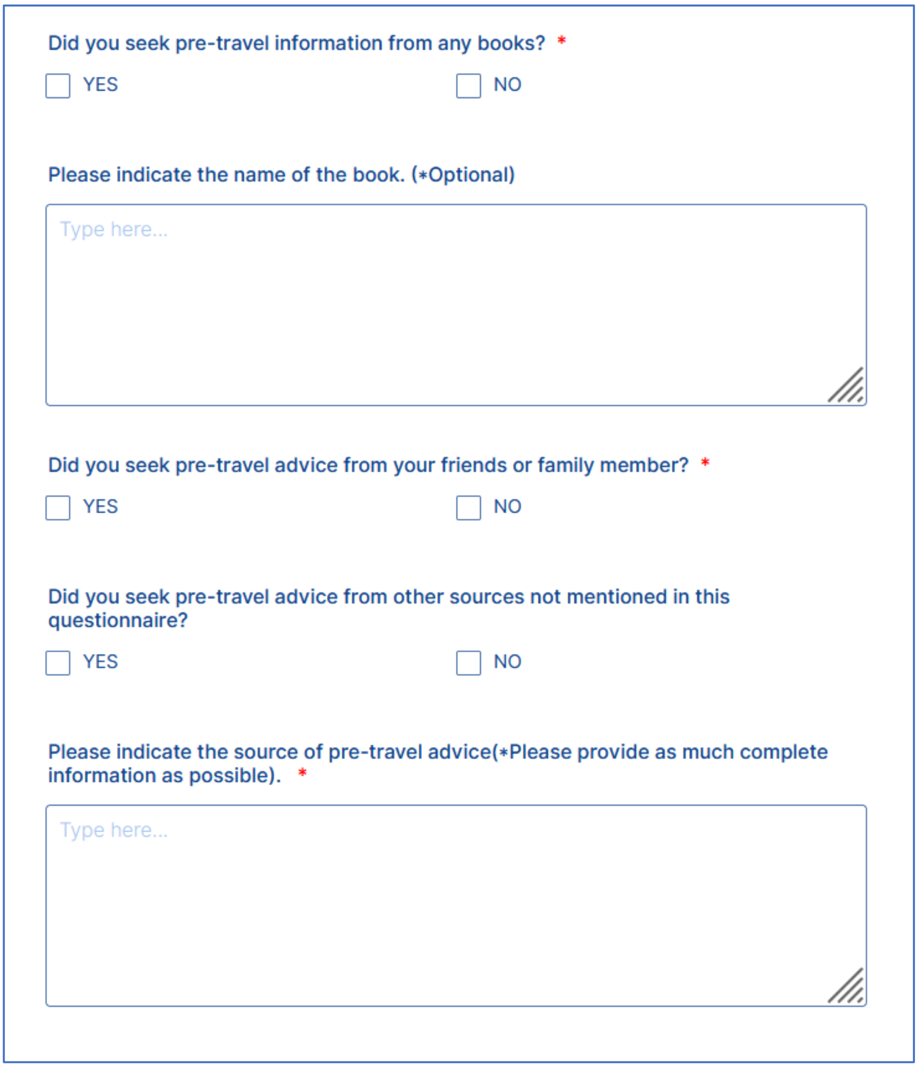

Did you seek pre-travel information from any books? *

☐ YES ☐ NO

Please indicate the name of the book. (*Optional)

Type here...

Did you seek pre-travel advice from your friends or family member? *

☐ YES ☐ NO

Did you seek pre-travel advice from other sources not mentioned in this questionnaire?

☐ YES ☐ NO

Please indicate the source of pre-travel advice(*Please provide as much complete information as possible). *

Type here...

Figure S2: Presentation of the questionnaire (cont.)

.

Section 2.2: Source of pre-travel health advice.

"Did you receive any of these vaccines before coming to Thailand?"

Please select "YES" for each vaccine received before coming to Thailand this history included childhood vaccines, adult routine vaccines, and recommended travel vaccines.

COVID-19 vaccine *

☐ YES ☐ NO
☐ NOT SURE

Did you receive the COVID-19 vaccine specifically for this trip? *

☐ YES ☐ NO

Dengue vaccine *

☐ YES ☐ NO
☐ NOT SURE

Did you receive the Dengue vaccine specifically for this trip? *

☐ YES ☐ NO

Hepatitis A vaccine *

☐ YES ☐ NO
☐ NOT SURE

Did you receive the Hepatitis A vaccine specifically for this trip? *

☐ YES ☐ NO

Hepatitis B vaccine *

☐ YES ☐ NO
☐ NOT SURE

Did you receive the Hepatitis B vaccine specifically for this trip? *

☐ YES ☐ NO

Human papilloma visus (HPV) vaccine *

☐ YES ☐ NO
☐ NOT SURE

Did you receive the Human papilloma visus (HPV) vaccine specifically for this trip? *

☐ YES ☐ NO

Figure S2: Presentation of the questionnaire (cont.)

Influenza vaccine *

☐ YES ☐ NO

☐ NOT SURE

Did you receive the Influenza vaccine specifically for this trip? *

☐ YES ☐ NO

Japanese encephalitis vaccine *

☐ YES ☐ NO

☐ NOT SURE

Did you receive the Japanese encephalitis vaccine specifically for this trip? *

☐ YES ☐ NO

Measles/Mumps/Rubella vaccine *

☐ YES ☐ NO

☐ NOT SURE

Did you receive the Measles/Mumps/Rubella vaccine specifically for this trip? *

☐ YES ☐ NO

Rabies vaccine *

☐ YES ☐ NO

☐ NOT SURE

Did you receive the Rabies vaccine specifically for this trip? *

☐ YES ☐ NO

A booster dose of Tetanus/diptheria/Pertusis vaccine, either Tdap or Td *

☐ YES ☐ NO

☐ NOT SURE

Did you receive the Tdap or Td vaccine specifically for this trip? *

☐ YES ☐ NO

Did you receive other vaccines specifically for this trip? *

☐ YES ☐ NO

Figure S2: Presentation of the questionnaire (cont.)

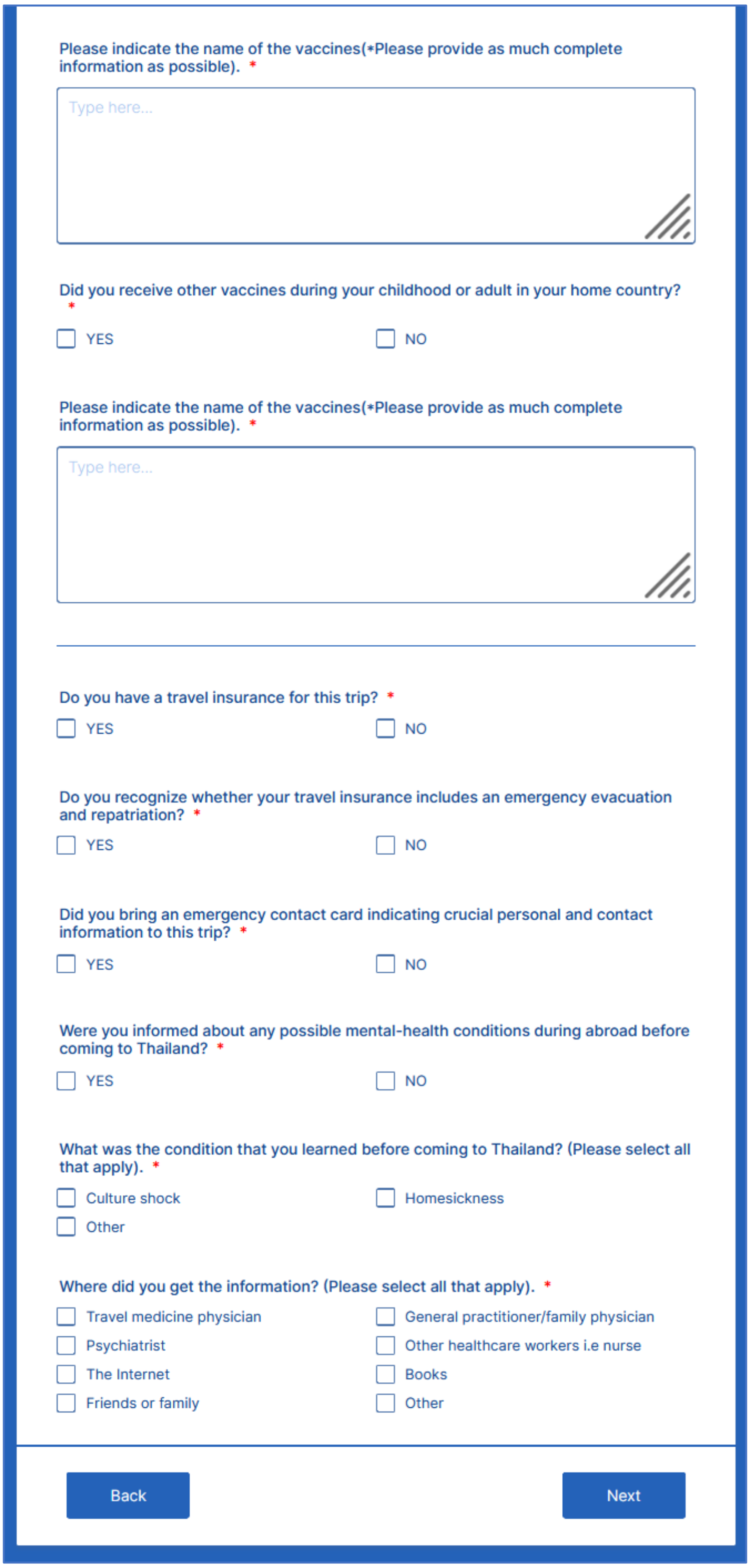

Please indicate the name of the vaccines(*Please provide as much complete information as possible). *

Type here...

Did you receive other vaccines during your childhood or adult in your home country? *

☐ YES ☐ NO

Please indicate the name of the vaccines(*Please provide as much complete information as possible). *

Type here...

Do you have a travel insurance for this trip? *

☐ YES ☐ NO

Do you recognize whether your travel insurance includes an emergency evacuation and repatriation? *

☐ YES ☐ NO

Did you bring an emergency contact card indicating crucial personal and contact information to this trip? *

☐ YES ☐ NO

Were you informed about any possible mental-health conditions during abroad before coming to Thailand? *

☐ YES ☐ NO

What was the condition that you learned before coming to Thailand? (Please select all that apply). *

☐ Culture shock ☐ Homesickness

☐ Other

Where did you get the information? (Please select all that apply). *

☐ Travel medicine physician ☐ General practitioner/family physician

☐ Psychiatrist ☐ Other healthcare workers i.e nurse

☐ The Internet ☐ Books

☐ Friends or family ☐ Other

Back Next

Figure S2: Presentation of the questionnaire (cont.)

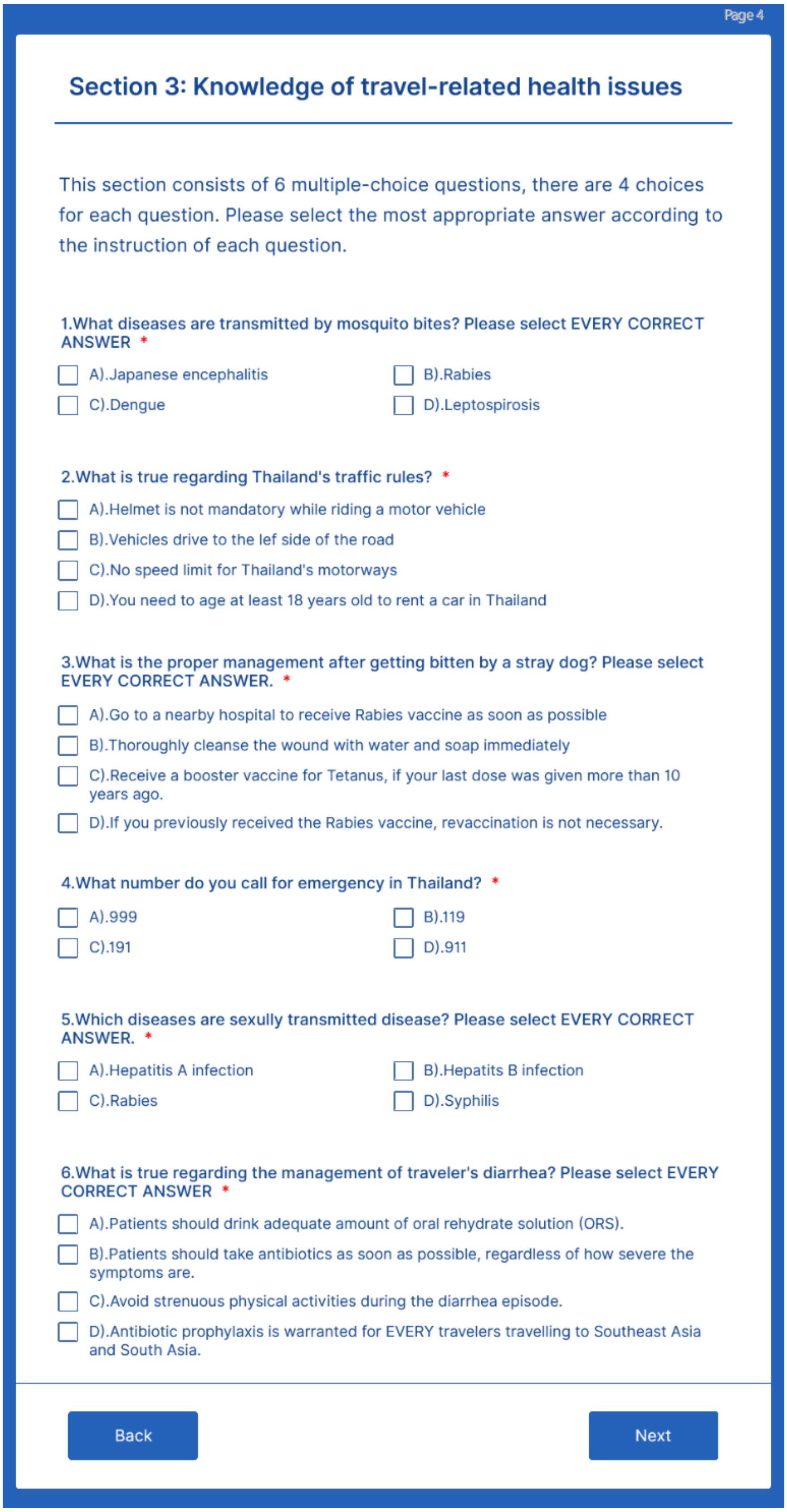

Page 4

Section 3: Knowledge of travel-related health issues

This section consists of 6 multiple-choice questions, there are 4 choices for each question. Please select the most appropriate answer according to the instruction of each question.

1.What diseases are transmitted by mosquito bites? Please select EVERY CORRECT ANSWER *

- [ ] A).Japanese encephalitis
- [ ] B).Rabies
- [ ] C).Dengue
- [ ] D).Leptospirosis

2.What is true regarding Thailand's traffic rules? *

- [ ] A).Helmet is not mandatory while riding a motor vehicle
- [ ] B).Vehicles drive to the lef side of the road
- [ ] C).No speed limit for Thailand's motorways
- [ ] D).You need to age at least 18 years old to rent a car in Thailand

3.What is the proper management after getting bitten by a stray dog? Please select EVERY CORRECT ANSWER. *

- [ ] A).Go to a nearby hospital to receive Rabies vaccine as soon as possible
- [ ] B).Thoroughly cleanse the wound with water and soap immediately
- [ ] C).Receive a booster vaccine for Tetanus, if your last dose was given more than 10 years ago.
- [ ] D).If you previously received the Rabies vaccine, revaccination is not necessary.

4.What number do you call for emergency in Thailand? *

- [ ] A).999
- [ ] B).119
- [ ] C).191
- [ ] D).911

5.Which diseases are sexully transmitted disease? Please select EVERY CORRECT ANSWER. *

- [ ] A).Hepatitis A infection
- [ ] B).Hepatits B infection
- [ ] C).Rabies
- [ ] D).Syphilis

6.What is true regarding the management of traveler's diarrhea? Please select EVERY CORRECT ANSWER *

- [ ] A).Patients should drink adequate amount of oral rehydrate solution (ORS).
- [ ] B).Patients should take antibiotics as soon as possible, regardless of how severe the symptoms are.
- [ ] C).Avoid strenuous physical activities during the diarrhea episode.
- [ ] D).Antibiotic prophylaxis is warranted for EVERY travelers travelling to Southeast Asia and South Asia.

Back Next

Figure S2: Presentation of the questionnaire (cont.)

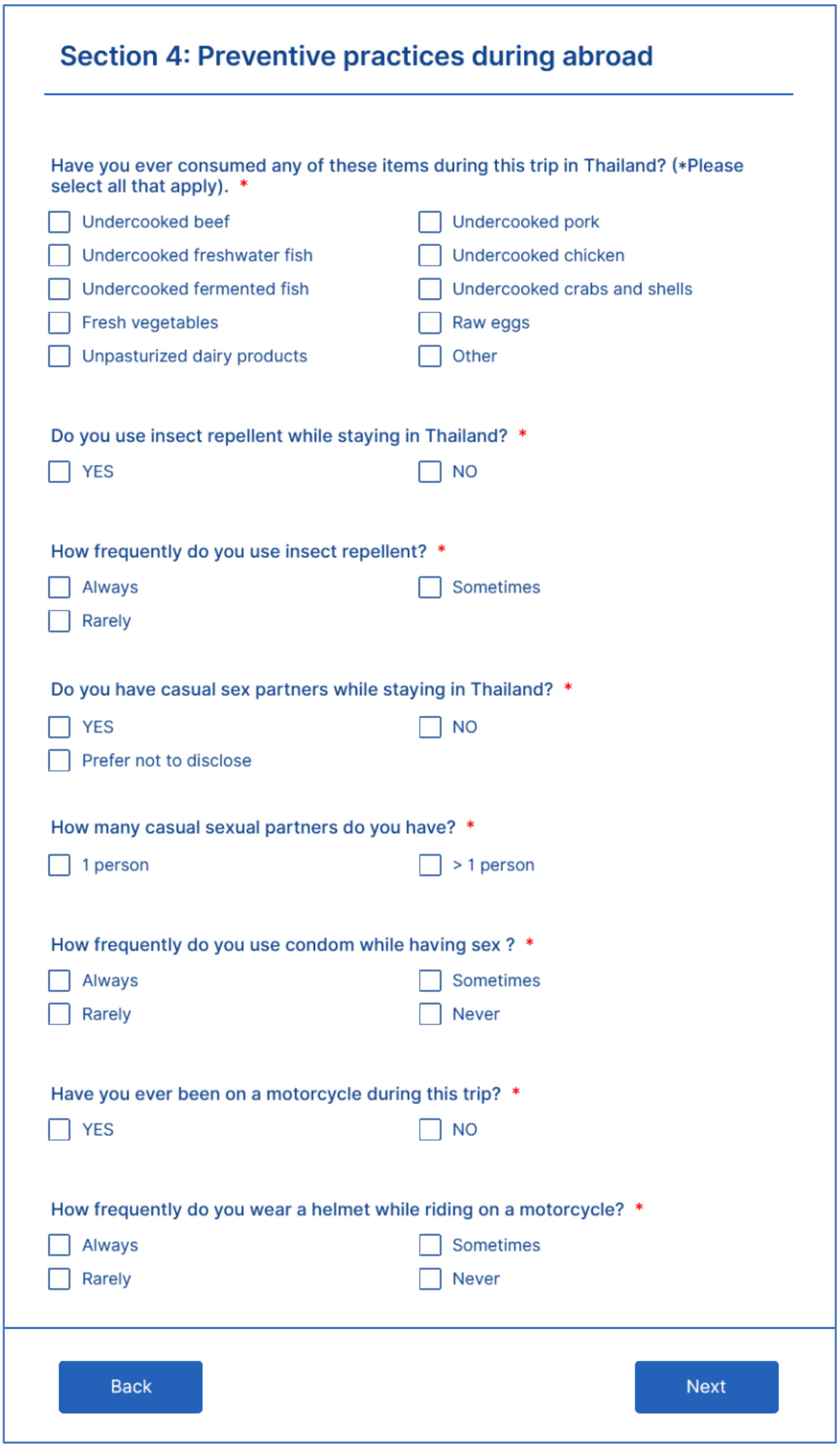

Section 4: Preventive practices during abroad

Have you ever consumed any of these items during this trip in Thailand? (*Please select all that apply). *

- ☐ Undercooked beef
- ☐ Undercooked pork
- ☐ Undercooked freshwater fish
- ☐ Undercooked chicken
- ☐ Undercooked fermented fish
- ☐ Undercooked crabs and shells
- ☐ Fresh vegetables
- ☐ Raw eggs
- ☐ Unpasturized dairy products
- ☐ Other

Do you use insect repellent while staying in Thailand? *

- ☐ YES
- ☐ NO

How frequently do you use insect repellent? *

- ☐ Always
- ☐ Sometimes
- ☐ Rarely

Do you have casual sex partners while staying in Thailand? *

- ☐ YES
- ☐ NO
- ☐ Prefer not to disclose

How many casual sexual partners do you have? *

- ☐ 1 person
- ☐ > 1 person

How frequently do you use condom while having sex ? *

- ☐ Always
- ☐ Sometimes
- ☐ Rarely
- ☐ Never

Have you ever been on a motorcycle during this trip? *

- ☐ YES
- ☐ NO

How frequently do you wear a helmet while riding on a motorcycle? *

- ☐ Always
- ☐ Sometimes
- ☐ Rarely
- ☐ Never

Back Next

Figure S2: Presentation of the questionnaire (cont.)

Section 5: Travel-related health problems in Thailand

Please select **"YES"** for the illness that you experienced while studying in Thailand. (Please select all that apply.) In case that you did not experience any illnesses, please select "NO" and click the button "Next".

Have you ever experienced any illnesses during this trip in Thailand? *

- ☐ YES
- ☐ NO

Acute diarrhea (Passing loose or water stool 3 or more episodes per day, or passing mucous/bloody stool). *

- ☐ YES
- ☐ NO

When did the symptom occur? (In case there was more than 1 episode, please answer for the only 1 clinically significant episode). *

- ☐ < 3 months after arrival
- ☐ 3-6 months after arrival
- ☐ >6 months after arrival

How did you seek the treatment for the illness? *

- ☐ Spontaneously resolved without any treatments
- ☐ Self-medicated
- ☐ Treated by a medical doctor as an out-patient case.
- ☐ Hospitalized

Acute febrile illness. *

- ☐ YES
- ☐ NO

When did the symptom occur? (In case there was more than 1 episode, please answer for the only 1 clinically significant episode). *

- ☐ < 3 months after arrival
- ☐ 3-6 months after arrival
- ☐ >6 months after arrival

How did you seek the treatment for the illness? *

- ☐ Spontaneously resolved without any treatments
- ☐ Self-medicated
- ☐ Treated by a medical doctor as an out-patient case.
- ☐ Hospitalized

Did you know the definite diagnosis of you acute febrile illness? *

- ☐ YES
- ☐ NO

Please indicate the definite diagnosis (*Please provide complete information as much as possible). *

[ ]

Figure S2: Presentation of the questionnaire (cont.)

Respiratory tract diseases. *

☐ YES ☐ NO

When did the symptom occur? (In case there was more than 1 episode, please answer for the only 1 clinically significant episode). *

☐ < 3 months after arrival ☐ 3-6 months after arrival

☐ >6 months after arrival

How did you seek the treatment for the illness? *

☐ Spontaneously resolved without any treatments ☐ Self-medicated

☐ Treated by a medical doctor as an out-patient case. ☐ Hospitalized

Did you know the definite diagnosis of you illness? *

☐ YES ☐ NO

Please indicate the definite diagnosis (*Please provide complete information as much as possible). *

[ ]

Dermatological diseases. *

☐ YES ☐ NO

When did the symptom occur? (In case there was more than 1 episode, please answer for the only 1 clinically significant episode). *

☐ < 3 months after arrival ☐ 3-6 months after arrival

☐ >6 months after arrival

How did you seek the treatment for the illness? *

☐ Spontaneously resolved without any treatments ☐ Self-medicated

☐ Treated by a medical doctor as an out-patient case. ☐ Hospitalized

Did you know the definite diagnosis of you illness? *

☐ YES ☐ NO

Please indicate the definite diagnosis (*Please provide complete information as much as possible). *

[ ]

Figure S2: Presentation of the questionnaire (cont.)

Animal contact (Including getting licked, scratched or bitten). *

☐ YES ☐ NO

What kind of animal that you got licked, scratched or bitten? *

☐ Dog ☐ Cat

☐ Monkey ☐ Other

When did the incident occur? *

☐ < 3 months after arrival ☐ 3-6 months after arrival

☐ >6 months after arrival

Did you recieve Rabies vaccination after the animal contact? *

☐ YES ☐ NO

Traffic-related physical injuries *

☐ YES ☐ NO

Please indicate how the accident happened. (*Please provide complete information as much as possible). *

Type here...

Please describe the injuries that you suffered from the accident.(*Please provide complete information as much as possible). *

Type here...

When did the injury occur? *

☐ < 3 months after arrival ☐ 3-6 months after arrival

☐ >6 months after arrival

How did you seek the treatment for the injury? *

☐ Spontaneously resolved without any treatments ☐ Self-medicated

☐ Treated by a medical doctor as an out-patient case. ☐ Hospitalized

Figure S2: Presentation of the questionnaire (cont.)

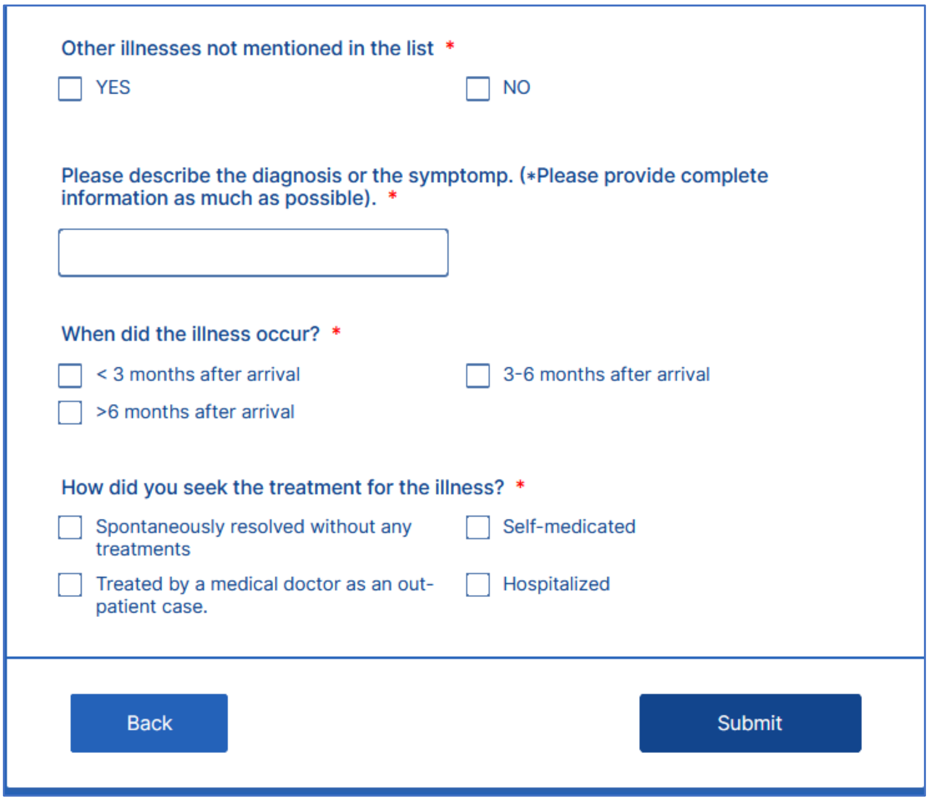

Other illnesses not mentioned in the list *

☐ YES ☐ NO

Please describe the diagnosis or the symptomp. (*Please provide complete information as much as possible). *

When did the illness occur? *

☐ < 3 months after arrival ☐ 3-6 months after arrival

☐ >6 months after arrival

How did you seek the treatment for the illness? *

☐ Spontaneously resolved without any treatments ☐ Self-medicated

☐ Treated by a medical doctor as an out-patient case. ☐ Hospitalized

Back Submit

Figure S2: Presentation of the questionnaire (cont.)

.
